# Supplementary material for: Interventions Based on Mind–Body Therapies for the Improvement of Attention-Deficit/Hyperactivity Disorder Symptoms in Youth: A Systematic Review
Source: Medicina (Kaunas). 2019 Jun 30;55(7):325. doi: 10.3390/medicina55070325 (PMC6680862; doi:10.3390/medicina55070325)
Supplement: Supplementary file 1 [file medicina-55-00325-s001.pdf]

Supplementary Table 1. PRISMA-P 2015 Checklist

PRISMA-P 2015 Checklist

Title: Interventions based on Mind-Body Therapies for the improvement of attention-deficit/ hyperactivity disorder symptoms in youth: a systematic review

Section/topic	#	Checklist item	Information reported		Line number(s)
			Yes	No	
**ADMINISTRATIVE INFORMATION**					
**Title**					
Identification	1a	Identify the report as a protocol of a systematic review	☒	☐	3
Update	1b	If the protocol is for an update of a previous systematic review, identify as such	☐	☒	
**Registration**	2	If registered, provide the name of the registry (e.g., PROSPERO) and registration number in the Abstract	☐	☒	
**Authors**					
Contact	3a	Provide name, institutional affiliation, and e-mail address of all protocol authors; provide physical mailing address of corresponding author	☒	☐	8-17
Contributions	3b	Describe contributions of protocol authors and identify the guarantor of the review	☒	☐	19-20
**Amendments**	4	If the protocol represents an amendment of a previously completed or published protocol, identify as such and list changes; otherwise, state plan for documenting important protocol amendments	☐	☒	
**Support**					
Sources	5a	Indicate sources of financial or other support for the review	☐	☐	NA
Sponsor	5b	Provide name for the review funder and/or sponsor	☐	☐	NA

Section/topic	#	Checklist item	Information reported		Line number(s)
			Yes	No	
Role of sponsor/funder	5c	Describe roles of funder(s), sponsor(s), and/or institution(s), if any, in developing the protocol	☐	☐	NA
**INTRODUCTION**					
**Rationale**	6	Describe the rationale for the review in the context of what is already known	☒	☐	45-61
**Objectives**	7	Provide an explicit statement of the question(s) the review will address with reference to participants, interventions, comparators, and outcomes (PICO)	☒	☐	130-139
**METHODS**					
**Eligibility criteria**	8	Specify the study characteristics (e.g., PICO, study design, setting, time frame) and report characteristics (e.g., years considered, language, publication status) to be used as criteria for eligibility for the review	☒	☐	130-139
**Information sources**	9	Describe all intended information sources (e.g., electronic databases, contact with study authors, trial registers, or other grey literature sources) with planned dates of coverage	☒	☐	130-139 SF1
**Search strategy**	10	Present draft of search strategy to be used for at least one electronic database, including planned limits, such that it could be repeated	☒	☐	SF1
STUDY RECORDS					
Data management	11a	Describe the mechanism(s) that will be used to manage records and data throughout the review	☒	☐	148-164 Fig 1
Selection process	11b	State the process that will be used for selecting studies (e.g., two independent reviewers) through each phase of the review (i.e., screening, eligibility, and inclusion in meta-analysis)	☒	☐	148-176

Section/topic	#	Checklist item	Information reported		Line number(s)
			Yes	No	
Data collection process	11c	Describe planned method of extracting data from reports (e.g., piloting forms, done independently, in duplicate), any processes for obtaining and confirming data from investigators	☒	☐	166-176
**Data items**	12	List and define all variables for which data will be sought (e.g., PICO items, funding sources), any pre-planned data assumptions and simplifications	☒	☐	130-139<br>SF1
**Outcomes and prioritization**	13	List and define all outcomes for which data will be sought, including prioritization of main and additional outcomes, with rationale	☒	☐	126-129
**Risk of bias in individual studies**	14	Describe anticipated methods for assessing risk of bias of individual studies, including whether this will be done at the outcome or study level, or both; state how this information will be used in data synthesis	☒	☐	177-197
DATA					
**Synthesis**	15a	Describe criteria under which study data will be quantitatively synthesized	☐	☐	NA
	15b	If data are appropriate for quantitative synthesis, describe planned summary measures, methods of handling data, and methods of combining data from studies, including any planned exploration of consistency (e.g., I^2, Kendall's tau)	☐	☐	NA
	15c	Describe any proposed additional analyses (e.g., sensitivity or subgroup analyses, meta-regression)	☐	☐	NA
	15d	If quantitative synthesis is not appropriate, describe the type of summary planned	☐	☐	NA
**Meta-bias(es)**	16	Specify any planned assessment of meta-bias(es) (e.g., publication bias across studies, selective reporting within studies)	☐	☐	NA

Section/topic	#	Checklist item	Information reported		Line number(s)
			Yes	No	
**Confidence in cumulative evidence**	17	Describe how the strength of the body of evidence will be assessed (e.g., GRADE)	☐	☐	NA

NA not applicable

SF1 supplementary file 1

Fig. 1 Figure 1

Supplementary file 1. Electronic search: database and terms included.

The electronic search was conducted from 1st January 2000 to 31th December 2018. A PICOS approach was used for framing the research question and the evidence search. **P**ARTICIPANTS: *Child* OR adolescent* OR young* OR youth.* **I**NTERVENTIONS: *Yoga OR yogic OR meditation OR Tai Chi OR mindfulness OR mindful OR mindfulness-based OR mind-body OR relaxation OR zen.* **C**OMPARISONS: Not applicable. **O**UTCOMES: Medical condition terms (*ADHD OR attention deficit OR attention-deficit OR hyperkinetic syndrome OR hyperkinetic disorder).* **S**TUDY DESIGN: *intervention OR program OR therapy OR training OR school based intervention.* Additional filters: All database, builder terms: Title/abstract for PUBMED, TOPIC for WOS, and abstract for PsycINFO and EBSCOhost.

The following terms were used for each category:

a) **Pubmed:** (from 01-01-2000 to 2018)

((((Child*[Title/Abstract] OR adolescent*[Title/Abstract] OR young*[Title/Abstract] OR youth[Title/Abstract])) AND (Yoga[Title/Abstract] OR yogic[Title/Abstract] OR meditation[Title/Abstract] OR Tai Chi[Title/Abstract] OR mindfulness[Title/Abstract] OR mindful[Title/Abstract] OR mindfulness-based[Title/Abstract] OR mind-body[Title/Abstract] OR relaxation[Title/Abstract] OR zen[Title/Abstract])) AND (ADHD[Title/Abstract] OR attention deficit[Title/Abstract] OR attention-deficit[Title/Abstract] OR hyperkinetic syndrome[Title/Abstract] OR hyperkinetic disorder[Title/Abstract])) AND (intervention[Title/Abstract] OR program[Title/Abstract] OR therapy[Title/Abstract] OR training[Title/Abstract] OR school based intervention[Title/Abstract])

Additional filters: All database [builder term: Title/Abstract])

b) **WOS:** (from 2000 to 2018) main collection of Web of Science

TOPIC: (Child* OR adolescent* OR young* OR youth) *AND* TOPIC: (Yoga OR yogic OR meditation OR Tai Chi OR mindfulness OR mindful OR mindfulness-based OR mind-body OR relaxation OR zen) *AND* TOPIC: (ADHD OR attention deficit OR attention-deficit OR hyperkinetic syndrome OR hyperkinetic disorder) *AND* TOPIC: (intervention OR program OR therapy OR training OR school based intervention)

Additional filters: main collection of Web of Science [builder term: Topic])

c) PsycINFO: (from 01/01/2000 to 31/12/2018)

ab(Child OR adolescent* OR young* OR youth) AND ab(Yoga OR yogic OR meditation OR Tai Chi OR mindfulness OR mindful OR mindfulness-based OR mind-body OR relaxation OR zen) AND ab(ADHD OR attention deficit OR attention-deficit OR hyperkinetic syndrome OR hyperkinetic disorder) AND ab(intervention OR program OR therapy OR training OR school based intervention).*

Additional filters: [builder term: ab Abstract])

d) EBSCOhost: (from January 2000 to Dec 2018)

AB (Child OR adolescent* OR young* OR youth) AND AB (Yoga OR yogic OR meditation OR Tai Chi OR mindfulness OR mindful OR mindfulness-based OR mind-body OR relaxation OR zen) AND AB (ADHD OR attention deficit OR attention-deficit OR hyperkinetic syndrome OR hyperkinetic disorder) AND AB (intervention OR program OR therapy OR training OR school based intervention)*

Additional filters: [builder term: AB Abstract])

Supplementary Table 2. Quality Assessment of Controlled Intervention Studies.

	Gershy et al. 2017#	Chou et al. 2017*	Jensen et al. 2004#	Kiani et al. 2012#	Lo et al. 2017#	Behbahani 2018#
1. Was the study described as randomised, a randomised clinical trial, or an RCT?	Yes	Yes	Yes	Yes	Yes	Yes
1.1 Or did they describe it as cluster randomised?	NA	NA	NA	NA	NA	NA
2. Was the method of the randomisation adequate (i.e., use of randomly generated assignment)?	Yes	Yes	NA	Yes	Yes	Yes
3. Was the treatment allocation concealed (so that assignments could not be predicted)?	Yes	Yes	No	No	No	No
4.a) Were study participants blinded to the treatment-group assignments?	Yes	No	No	No	No	No
4. b) Were providers blinded to the treatment group assignments?	Yes	No	No	No	No	No
4.1 In case of cluster-randomisation: Was the recruitment of participants conducted by an individual independent of the trial?	NA	NA	NA	NA	NA	NA
5. Were the people blinded to the participant's group assignment?	Yes	No	No	No	No	No
6. Were the groups similar at baseline on important characteristics that could affect outcomes (i.e., demographics, risk-factors, co-morbid conditions)?	Yes	Yes	Yes	NA	Yes	Yes
6.1 In case of cluster randomisation: Did they use stratification or matched-pairs before randomisation to reduce baseline-imbalances?	NA	NA	NA	NA	NA	NA

7. Was the overall drop-out rate from the study at endpoint 20% or lower of the number allocated to treatment?	Yes	Yes	Yes	Yes	Yes	Yes
8. Was the differential drop-out rate (between treatment groups) at endpoint 15 percentage points or lower?	Yes	Yes	Yes	Yes	Yes	Yes
9. Was there high adherence to the intervention protocols for each treatment group?	Yes	Yes	Yes	NR	Yes	NR
10. Were other interventions avoided or similar in the groups (e.g., similar background treatments)?	NR	NR	NR	NR	NR	NR
12. Did the authors report the calculation of a sufficiently large sample size to be able to detect a difference in the main outcome between groups with at least 80% power?	No	No	No	No	No	No
12.1 a) In case of cluster-randomisation: Did they take clustering effects into account in their statistical analysis?	NA	NA	NA	NA	NA	NA
21.1 b) In case of cluster-randomisation: Did they consider intra-class-correlation regarding sample size calculation?	NA	NA	NA	NA	NA	NA
13. Were outcomes or analysed subgroups which were reported prespecified? (i.e., identified before analyses was conducted)?	Yes	Yes	Yes	Yes	Yes	Yes
14. Were all randomised participants analysed in the group to which they were originally assigned, i.e., did they use an intention-to-treat analysis?	Yes	Yes	Yes	Yes	Yes	Yes
Quality rating: (good, fair or poor)	fair	poor	poor	poor	poor	poor

[#] RCT study.

* Clinical trial study.

NA not applicable, *NR* not reported.

Supplementary Table 3. Quality Assessment for Before-After Studies (Pre-Po
Studies with No Control group).

	Haripras ad et al. 2013	Haydick y et al. 2015	Van der Oord et al. 2012	Zylow a et 2007
1. Was the study question or objective clearly stated?	Yes	Yes	Yes	Yes
2. Were eligibility/selection criteria for the study population prespecified and clearly described?	Yes	Yes	Yes	Yes
3. Were the participants in the study representative of those who were eligible for the test/service/intervention in the general or clinical population of interest?	No	No	No	No
4. Were all eligible participants that meet the prespecified entry criteria enrolled?	Yes	Yes	Yes	Yes
5. Was the sample size sufficiently large to provide confidence in the findings?	No	No	No	No
6. Was the test/service/intervention clearly described and delivered consistently across the study population?	Yes	Yes	Yes	Yes
7. Were the outcome measures prespecified, clearly defined, valid, reliable and assessed consistently across all study participants?	Yes	Yes	Yes	Yes
8. Were the people assessing the outcomes blinded to the participants' exposure/interventions?	No	No	No	No
9. Was the loss to follow-up after baseline 20% or less? Were those lost to follow-up accounted for in the analysis?	Yes	Yes	Yes	Yes
10. Did they use statistical methods that examined changes in outcome measures from before to after the intervention? Were statistical tests done that provided p values for the pre-to-post changes?	No	No	NR	Yes
11. Were outcome measures of interest taken multiple times before the intervention and multiples times after the intervention (i.e., did they use an interrupted time-series design)?	Yes	Yes	No	No
12. If the intervention was conducted at a group level (e.g., a whole hospital, a community, etc.) did the statistical analysis take into account the use of individual-level data to determine effects at the group level?	Yes	Yes	No	Yes
Quality rating: (good, fair or poor)	poor	poor	poor	poor

NR not reported
